# Supplementary material for: Reversal of Pulmonary Fibrosis: Human Umbilical Mesenchymal Stem Cells from Wharton’s Jelly versus Human-Adipose-Derived Mesenchymal Stem Cells
Source: Int J Mol Sci. 2023 Apr 8;24(8):6948. doi: 10.3390/ijms24086948 (PMC10139084; doi:10.3390/ijms24086948)
Supplement: Supplementary file 1 [file ijms-24-06948-s001.zip › ijms-2278088-supplementary.pdf]

Supplemental Figure Legends

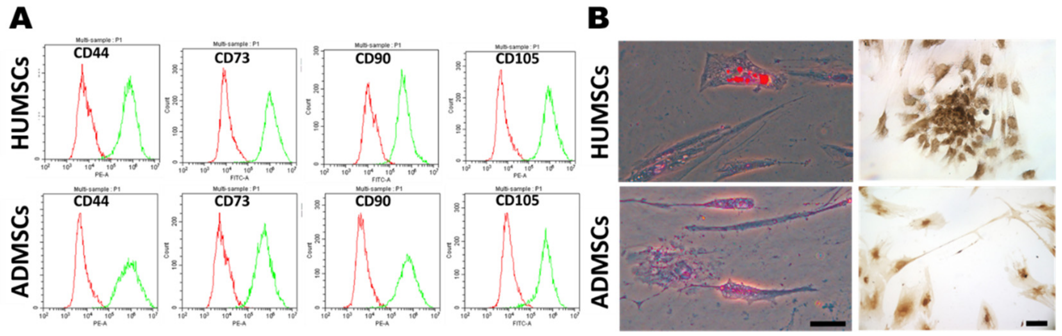

Supplemental Figure S1. Surface markers and differentiation ability of HUMSCs and ADMSCs *in vitro*.

(A) Flow cytometry analyses of surface markers of HUMSCs and ADMSCs *in vitro*. HUMSCs and ADMSCs both expressed similar surface markers with high levels of CD44, CD73, CD90, and CD105 expression. (B) Multipotency of HUMSCs and ADMSCs. HUMSCs and ADMSCs could differentiate into Oil Red O positive adipose cells (left panel) and NeuN positive neuronal cells (right panel) when cultured in differentiation medium. Scale bar: 100 µm

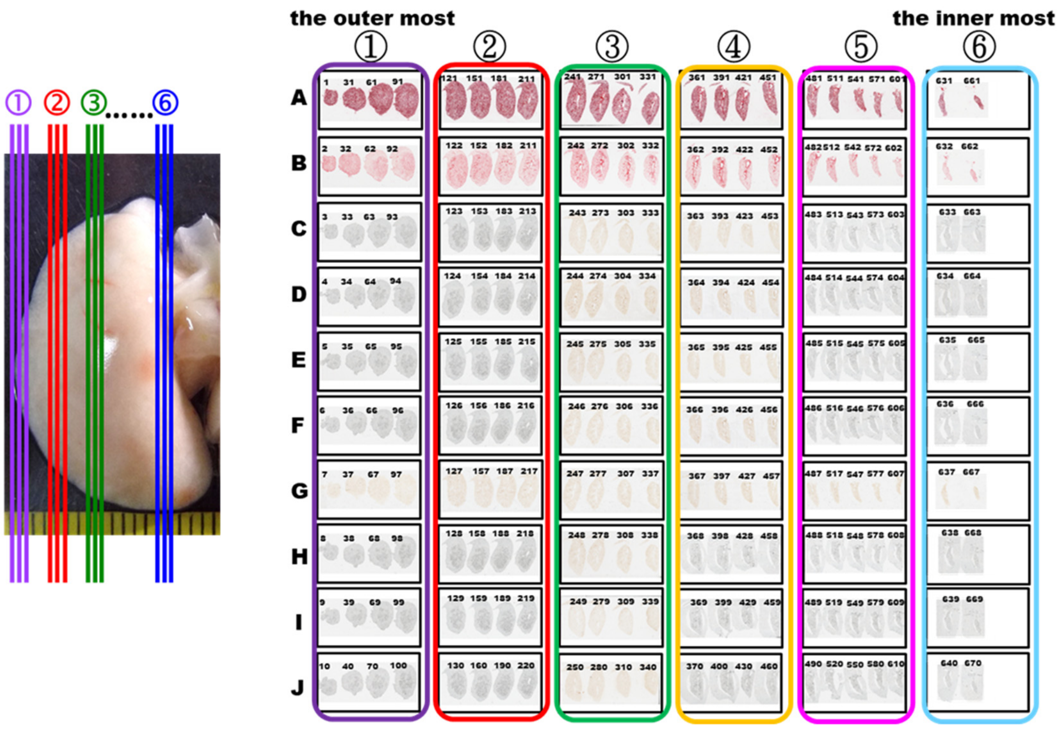

Supplemental Figure S2. An illustration of the sectioning procedure of paraffin-embedded lung tissues

A consecutive of 10 slices, numbered 1 through 10, were placed one by one on various glass slides (glass slides A to J) for either histochemical or immunochemical staining. On the 10 slides labeled A to J, lung slices numbered 1 to 10 were progressively arranged. Slices 11 through 30 were discarded (a total of 20 slices). Similar to this, slices 31 to 40 were put on slides A to J, and the following 20 slices (i.e., slices 41 to 60) were similarly thrown away. The identical procedures were carried out several times to thoroughly section the lung tissue. The left lung's inner area near the hilum is represented by Column ⑥ (blue frame), whereas the outermost portion of each lobe is shown by Column ① (purple frame). The slices in row A (numbered 1, 31, 61, 91, and 121…..661) were stained with H&E to examine the lung's histology; slices in row B (numbered 2, 32, 62, 92, 122…..662) were stained with Sirius Red to assess the degree of fibrosis; slices in row C (numbered 3, 33, 63, 93, 123…..663) were immunostained with anti-SMA to examine the changes in myofibroblasts; slices in row D (numbered 4, 34, 64, 94, 124…..664) were immunostained with anti-ED1 to label macrophages; slices in row E (numbered 5, 35, 65, 95, 125…..665) were immunostained with anti-CD86 antibody to label M1 macrophages; slices in row F (numbered 6, 36, 66, 96, 126…..666) were immunostained with anti-CD163 antibody to label M2 macrophages. The total number of lung slices ranged from 330 to 880, depending on the treatment group. The final rows (G–J) were kept around as backups.
